# Supplementary material for: Atlas of the anatomical localization of atypical chemokine receptors in healthy mice
Source: PLoS Biol. 2023 May 9;21(5):e3002111. doi: 10.1371/journal.pbio.3002111 (PMC10198502; doi:10.1371/journal.pbio.3002111)
Supplement: S1 Methods — (DOCX) [file pbio.3002111.s001.docx]

**Supporting methods**

**Creation of the** **gpr182-mCherry mouse line by Cas9/CRISPR 2-cell embryo microinjection.**

The coding sequence of gpr182 residing in a single exon of the gene was replaced with the mCherry orf using CRISPR/Cas9-mediated homologous recombination (HR). This led to the loss of Gpr182 gene function resulting in a reporter allele that expresses mCherry protein from the endogenous Gpr182 promoter. Two gRNA were designed to target the ATG and STOP codons of gpr182 respectively. The gRNA target sequences for ATG (**Met**) gctgggtatgactgac**atg**aggg and TAA (**stop**) tgcaattctgtagccagc**taa**gg codons (ngg PAM sequence underlined) were selected for optimal on target activity using the CRISPOR online tool (1). DNA double strand breaks generated by the Cas9RNPs of the two gRNAs resulted in the removal of the gpr182 orf. The resulting gap was repaired by HR using linear dsDNA template (GeneScript) comprised of the mCherry orf flanked by sequences complementary to the 5’UTR and 3’UTS regions of the Gpr182 gene, respectively. The linear dsDNA HR template sequence used is listed below (mCherry orf underlined):

ggatccaaagattttcccaggggcagctcagaggtgtgaggggtgggtgggtgtctgtctgtgtgtctgtgtatcccaaacctatgaccaaatagcaaggcagaacaggaacccctaatggagattgcactaaattagtaaaatctcaagagggaatcagaattagagaccactggggatgctagtaagctggaattgctgggaagaaaccttgttgggtacaagtaacaccaattttcctacaaaatgtctgttcttgggtcaaactaacgcagacataatctgagtcctaaacatgcttttctcaccttccctcagccagagagttcccccaggcccctgaggccttttagttggaggtatgggggcagacagaggctgccttctcagggactctctctctctttcttcttttccaaccctctcttctgtccaacagacccaagttggccctcatggtgagcaagggcgaggaggataacatggccatcatcaaggagttcatgcgcttcaaggtgcacatggagggctccgtgaacggccacgagttcgagatcgagggcgagggcgagggccgcccctacgagggcacccagaccgccaagctgaaggtgaccaagggtggccccctgcccttcgcctgggacatcctgtcccctcagttcatgtacggctccaaggcctacgtgaagcaccccgccgacatccccgactacttgaagctgtccttccccgagggcttcaagtgggagcgcgtgatgaacttcgaggacggcggcgtggtgaccgtgacccaggactcctccctgcaggacggcgagttcatctacaaggtgaagctgcgcggcaccaacttcccctccgacggccccgtaatgcagaagaagaccatgggctgggaggcctcctccgagcggatgtaccccgaggacggcgccctgaagggcgagatcaagcagaggctgaagctgaaggacggcggccactacgacgctgaggtcaagaccacctacaaggccaagaagcccgtgcagctgcccggcgcctacaacgtcaacatcaagttggacatcacctcccacaacgaggactacaccatcgtggaacagtacgaacgcgccgagggccgccactccaccggcggcatggacgagctgtacaagtaaggtagactctagcttcctccaccaacaagaaagttcagagggggatgcgagaggtctgtgggagggggtgggaaggactggcttgttcagggccaatttaagtatatcaaaatgttgctgtggggagagggaaacggttcgggaaggacagagaatggatctttccttgatagtacactatttgtttgggtactgatgtctaagggagccacaccggtggggcgtggggggtggggaagcgaaataaataaatcatagagacacccgtgctgggaatctctgagaagtgtgtacttccagctgggttgaggaaggcagggaggctaagggatgctctgcagagggaactagctgcccttgtttcataaatcaccctggccggaaggggaaaaaaaaccagccccacaggaaccttgcaaaactttccctttgtgtttgtctgcctgttgatattccaagttgtgaggacaagaggccagaggccaaggacccaggaggcctcggcaggacgttccctagctgtgcctcatccctaacccgggagtttgataaatcaagggggtggggattacagccagcaagctgggtcgtgggcaggcacccggcagctggctcaacaggatggatcc

Gene targeting was carried out by 2-cell embryo injections (2). Four weeks old C57BL/6J female mice underwent ovulation induction by i.p. injection of 5 IU equine chorionic gonadotrophin (PMSG; Folligon–InterVet), followed by i.p. injection of 5 IU human chorionic gonadotropin (Pregnyl–Essex Chemie) 48 h later. For the recovery of zygotes, C57BL/6J females were mated with mature males of the same strain immediately after the administration of human chorionic gonadotropin. All zygotes were collected from oviducts 24 h after the human chorionic gonadotropin injection and were then freed from any remaining cumulus cells by a 1–2 min treatment of 0.1% hyaluronidase (Sigma-Aldrich) dissolved in M2 medium (Sigma-Aldrich). Mouse embryos were cultured overnight in KSOM (Millipore) medium at 37°C and 5% CO_2_. Embryos that reached 2-cell stage the following morning were used for microinjections.

All microinjections were performed using a microinjection system comprised of an inverted microscope equipped with Nomarski optics (Nikon), a set of micromanipulators (Narashige), a FemtoJet microinjection unit (Eppendorf) and a micro ePore cell membrane penetrator (WPI).

Injection solution containing: Cas9 protein (IDT) 100ng/ul (60uM), cr:trcrRNA ATG (IDT) 50uM, cr:trcrRNA STOP (IDT) 50uM, dsDNA 20ng/ul was microinjected into the nuclei of both cells of the 2-cell embryos until 20-30% distension of the organelle was observed.

Embryos that survived the electroporation were transferred on the same day into the oviducts of 8–16-wk-old pseudopregnant Crl:CD1 (ICR) females (0.5 d used after coitus) that had been mated with sterile genetically vasectomized males (3) the day before embryo transfer. Pregnant females were allowed to deliver and raise their pups until weaning age and founders were identified by PCR analysis of genomic DNA obtained from biopsy samples.

Livers of ACKR4^GFP/GFP^ mice were removed, cut into ca. 4 mm cubes and fixed for 4 h in 2% PFA, embedded in OCT and snap-frozen and processed as above. Sections were stained with anti-CD31-PE (Biolegend) to visualize the liver sinusoids and the GFP signal was amplified with a primary rabbit anti-GFP followed by a secondary goat anti-rabbit labeled with AF488. Sections were imaged on a LSM800 (Zeiss).

References

(1) Concordet JP, Haeussler M. CRISPOR: intuitive guide selection for CRISPR/Cas9 genome editing experiments and screens. Nucleic Acids Res 2018 Jul 2;46(W1):W242-W245.

(2) Gu B, Posfai E, Rossant J. Efficient generation of targeted large insertions by microinjection into two-cell-stage mouse embryos. Nat Biotechnol 2018 Aug;36(7):632-7.

(3) Haueter S, Kawasumi M, Asner I, Brykczynska U, Cinelli P, Moisyadi S, et al. Genetic vasectomy-overexpression of Prm1-EGFP fusion protein in elongating spermatids causes dominant male sterility in mice. Genesis 2010 Mar;48(3):151-60.
