# Supplementary figures and images for: Atlas of the anatomical localization of atypical chemokine receptors in healthy mice
Source: PLoS Biol. 2023 May 9;21(5):e3002111. doi: 10.1371/journal.pbio.3002111 (PMC10198502; doi:10.1371/journal.pbio.3002111)

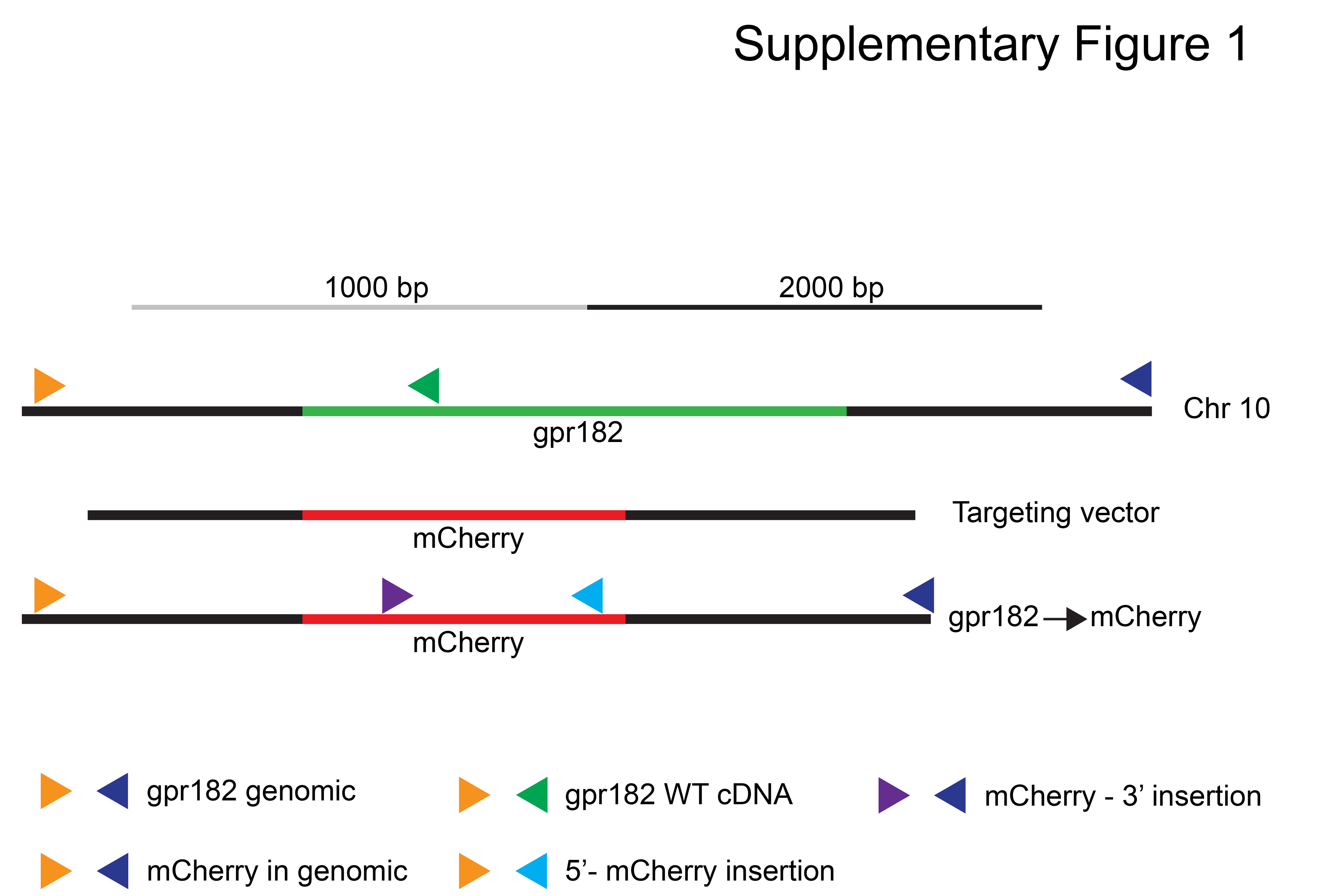

Supplement: S1 Fig — Schematic presentation showing the gene targeting strategy via CRISPR/Cas9 to insert mCherry into the gpr182 locus on chromosome 10. Triangles show sites of probe insertion for genotyping PCR. PCR, polymerase chain reaction. (TIF) [file pbio.3002111.s002.tif]

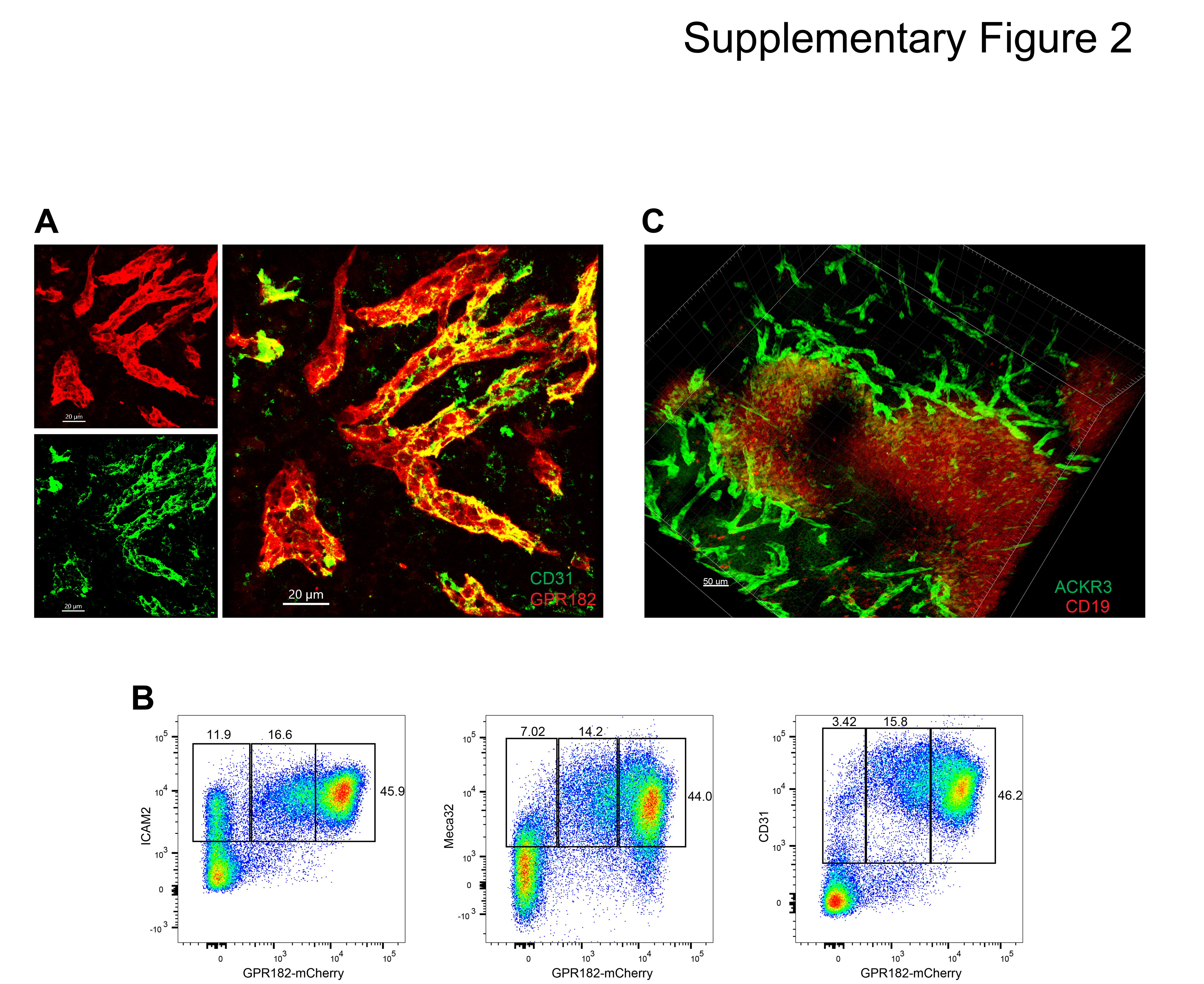

Supplement: S2 Fig — (A) Confocal images showing GPR182 (mCherry, red) colocalization with CD31+ vessels (green) in a cleared spleen (ACT-ECT) (scale bar = 20 μm). (B) Flow cytometry analysis on sorted GPR182-positive spleen endothelial cells stained for ICAM2, Meca32, and CD31. (C) ACKR3-expressing sinusoids (GFP, green) surround CD19+ B cell follicles (RFP, red; [29]). Yellow B cells (colocalization of red (RFP, CD19) and green (GFP, ACKR3)) are visible in the MZ (scale bar = 50 μm). FCS files and gating strategies are available in FlowRepository (S2B Fig). GFP, green fluorescent protein; MZ, marginal zone; RFP, red fluorescent protein. (TIF) [file pbio.3002111.s003.tif]

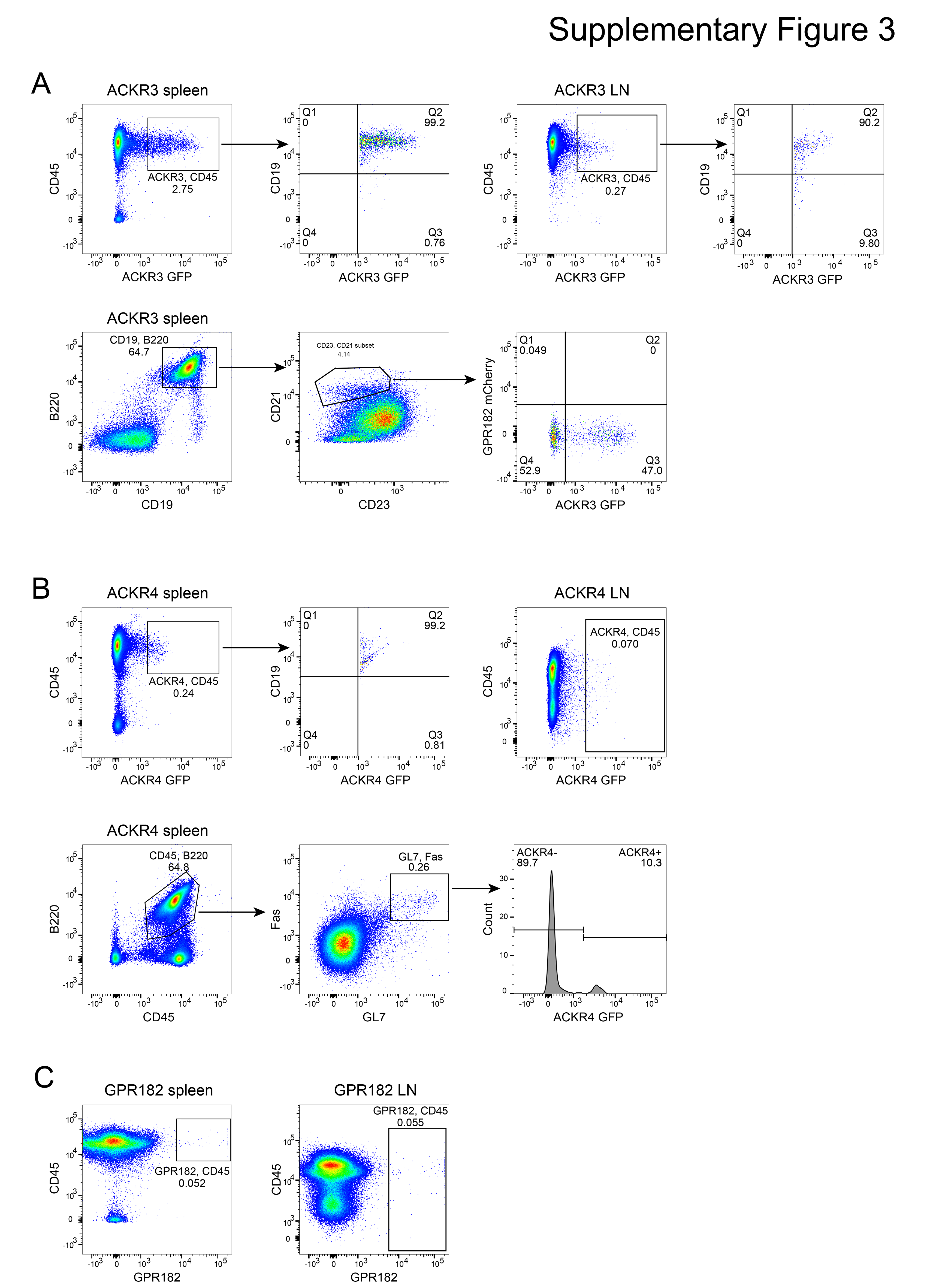

Supplement: S3 Fig — (A) Representative flow cytometry plots showing ACKR3-GFP expression in CD45+ cells, which were CD19+ in the spleen (two left upper panels), and in the LN (two right upper panels). Gating on CD19+ B220+, and CD21Hi CD23Low identifies MZB, of which 50% are ACKR3+ (lower panels). (B) Analysis of ACKR4-GFP expression in CD45+ cells (upper panels) identifies few cells in the spleen, which are CD19+, but almost none in the LN (right). Gating on CD45+ B220+, Fas+ GL7+ identifies GC B cells, of which 10% are ACKR4+ (lower panels). (C) GPR182-mCherry expression is absent in CD45+ cells in spleen (left) and LN (right). GFP and mCherry gates were set using a wild-type C57BL/6 mouse as negative control. FCS files and gating strategies are available in FlowRepository (S3A-S3C Fig). GC, germinal center; GFP, green fluorescent protein; LN, lymph node; MZB, marginal zone B cells. (TIF) [file pbio.3002111.s004.tif]

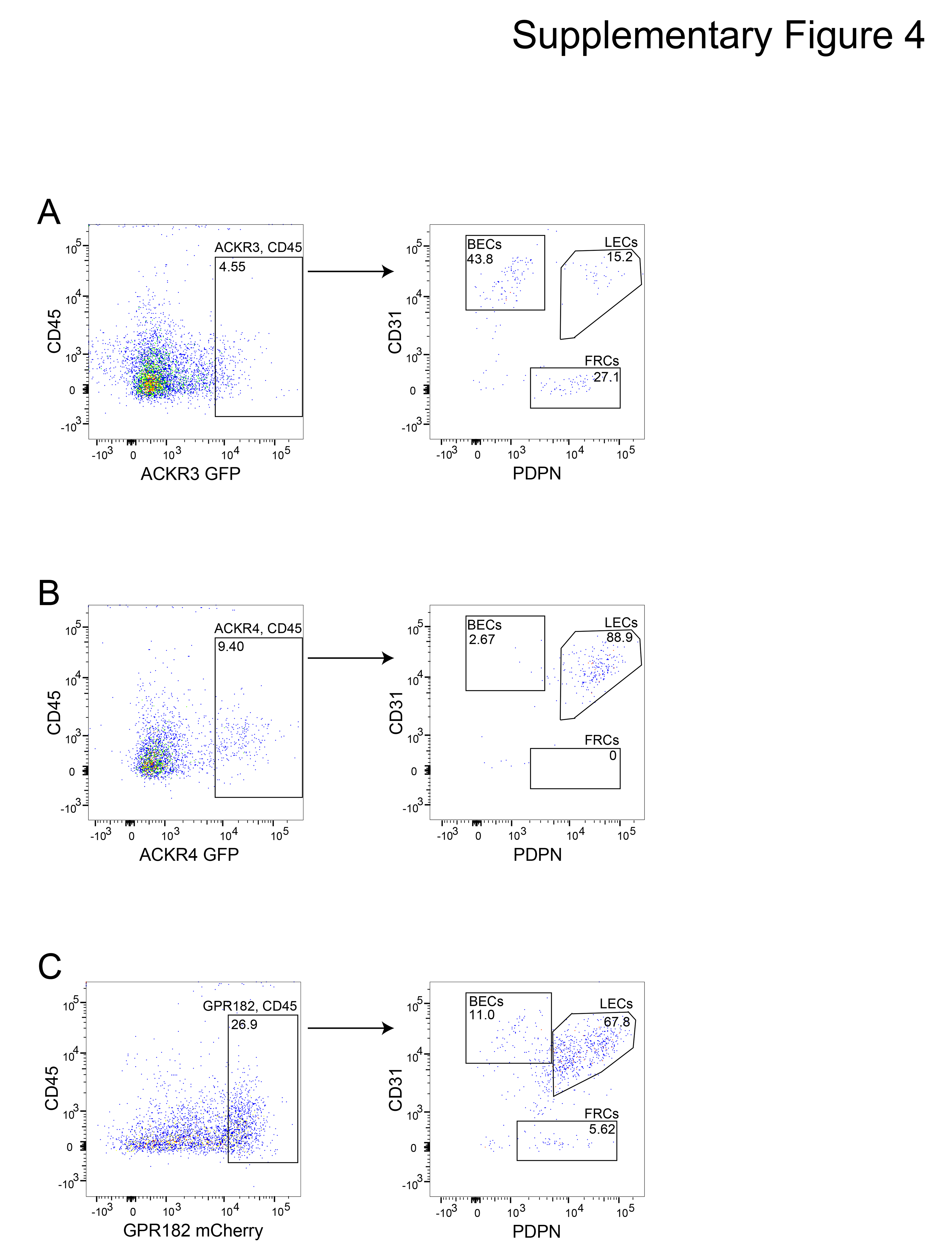

Supplement: S4 Fig — (A) Representative flow cytometry plots of CD45− cells, separated using negative selection on digested tissue, show the presence of few ACKR3+ CD45− cells, which are predominantly blood endothelial cells (right, CD31+ PDPN−). (B) Analysis of ACKR4-GFP expression in the CD45− fraction identifies expression on LECs (right, CD31+ PDPN+). (C) Analysis of GPR182-mCherry expression in the CD45− fraction identifies expression primarily on blood (right, CD31+ PDPN−) and lymphatic (CD31+ PDPN+) endothelial cells. GFP and mCherry gates were set using a wild-type C57BL/6 mouse as negative control. FCS files and gating strategies are available in FlowRepository (S4A-S4C Fig). GFP, green fluorescent protein; LEC, lymphatic endothelial cell; LN, lymph node. (TIF) [file pbio.3002111.s005.tif]

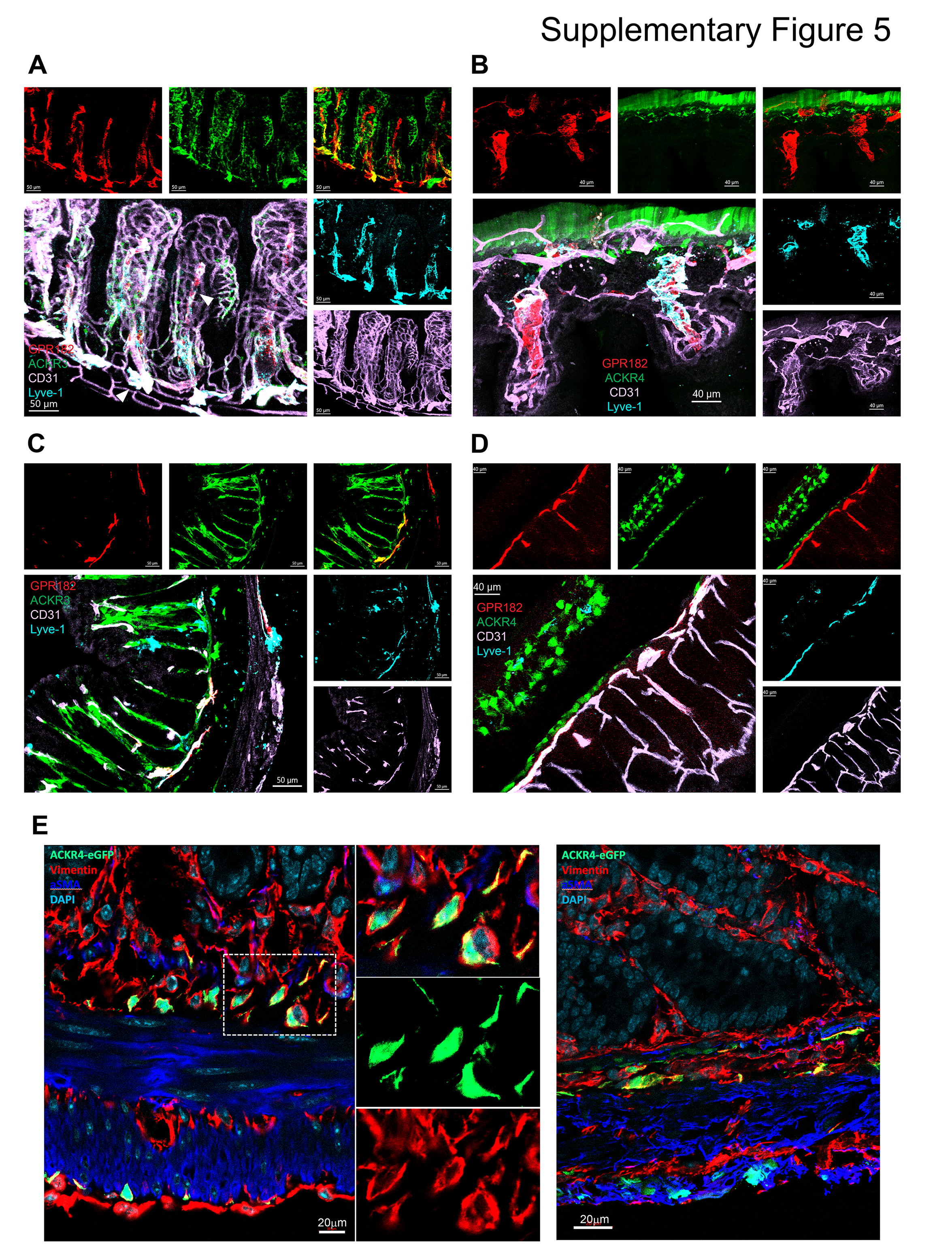

Supplement: S5 Fig — Magnified images of intestine from (A) ACKR3GFP/+ − GPR182mCherry/+ mouse, GPR182 (mCherry, red), ACKR3 (GFP, green), CD31 (pink), and Lyve-1 (cyan) (scale bar = 50 μm; arrow points to central lymphatic vessel), (B) ACKR4GFP/+ − GPR182mCherry/+ mouse, GPR182 (mCherry, red), ACKR4 (GFP, green), CD31 (pink), and Lyve-1 (cyan) (scale bar = 40 μm). (C) Magnified images of colon from ACKR3GFP/+ − GPR182mCherry/+ mouse, GPR182 (mCherry, red), ACKR3 (GFP, green), CD31 (pink), and Lyve-1 (cyan) (scale bar = 50 μm), and (D) ACKR4GFP/+ − GPR182mCherry/+ mouse, GPR182 (mCherry, red), ACKR4 (GFP, green), CD31 (pink), and Lyve-1 (cyan) (scale bar = 40 μm). (E) Immunofluorescence image of a section from small intestine (left image, with corresponding magnifications, including single channels) and colon (right image) from an ACKR4GFP/GFP mouse, showing ACKR4-expressing cells (GFP, green) and vimentin-positive cells (red), cell nuclei DAPI (cyan), and αSMA (blue) (scale bar = 20 μm). GFP, green fluorescent protein. (TIF) [file pbio.3002111.s006.tif]

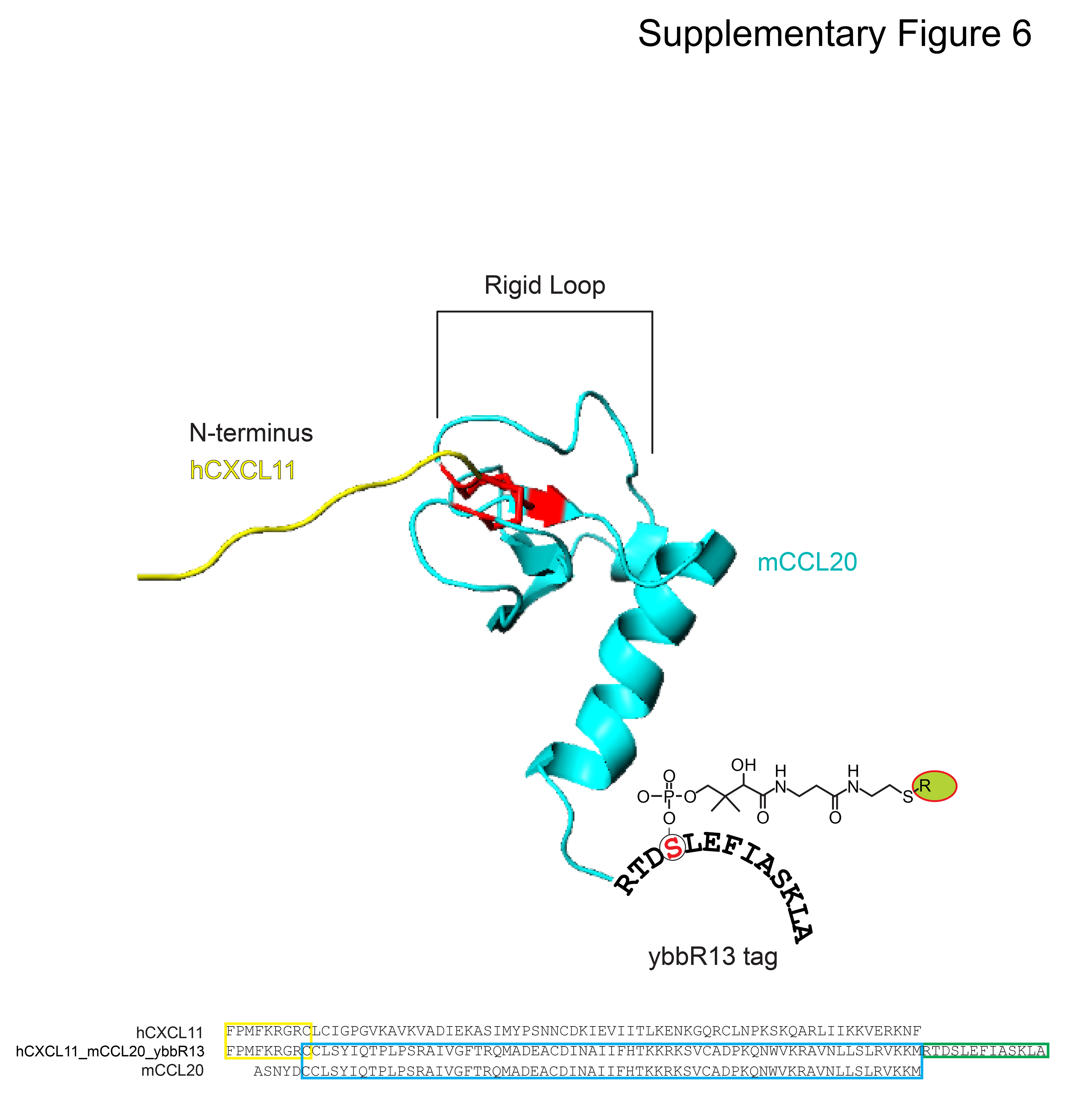

Supplement: S6 Fig — In silico structure of CXCL11_20, containing hCXCL11 (yellow) at the N-terminus and the body of mCCL20 (cyan). A ybbR13 tag is present at the C-terminus to allow for site specific labeling with phosphopantetheinyl transferase and a fluorescent labeled Co-enzyme A. (TIF) [file pbio.3002111.s007.tif]

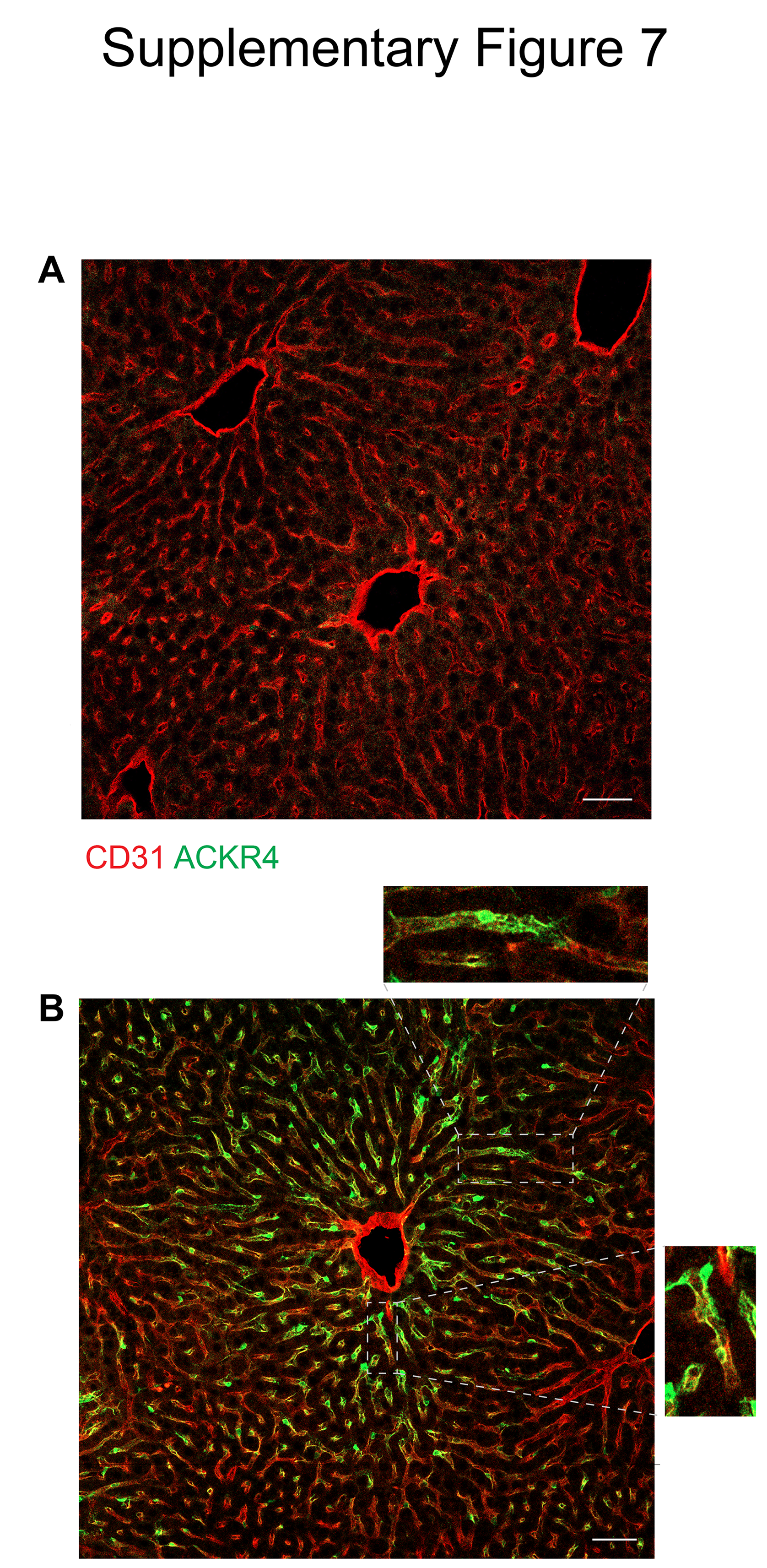

Supplement: S7 Fig — (A) Confocal images of ACKR4GFP/+ mouse liver sinusoids (CD31, red), without amplification of the eGFP signal. Scale bar = 20 μm. (B) Confocal images of ACKR4GFP/+ mouse liver sinusoids (CD31, red), showing expression of ACKR4 (anti-eGFP, green) at the segments proximal to the central vein, and gradually diminishing towards the periphery of the lobule. Scale bar = 20 μ. meGFP, enhanced green fluorescent protein. (TIF) [file pbio.3002111.s008.tif]
