# Supplementary material for: Atlas of the anatomical localization of atypical chemokine receptors in healthy mice
Source: PLoS Biol. 2023 May 9;21(5):e3002111. doi: 10.1371/journal.pbio.3002111 (PMC10198502; doi:10.1371/journal.pbio.3002111)
Supplement: Attachment — Submitted filename: Reply to reviewers comments.pdf [file pbio.3002111.s009.pdf]

REVIEWS:

Reviewer #1: The manuscript "Expression atlas of the anatomical localization of atypical chemokine receptors" by Melgrati et al. was submitted as a Methods and Resources article to PLOS Biology. It uses mice expressing fluorescent reporter proteins from genes of atypical chemokine receptors and intravenous injection of semi-specific chemokine chimeras to map the anatomical localization of atypical chemokine receptors in immune organs and some other tissues.

Criteria:

According to the PLOS Biology criteria, Methods and Resources articles need to report a novel method or improvements to current methodologies that significantly outperform the state-of-the-art methods or that show the potential to address, for the first time, a pressing biological question. Ideally, these Methods should be of broad interest. Furthermore, Resources are required to be truly exceptional to spur future research.

Criticism:

The use of fluorescent reporter mice to map the anatomical localization of the expression of a gene of interest is a standard technique and, as such, fails to meet the criteria for Methods and Resources articles. Furthermore, the anatomical localization of atypical chemokine receptors in immune organs and other organs has been reported before based on fluorescent reporter mice and other techniques. This leaves intravenous injection of semi-specific chemokine chimeras to reveal scavenging activity of chemokine receptors. However, this technique has several shortcomings. The i.v.-injected chemokine has access only to receptors that face the circulation. Polarized cells in which the receptor of interest is targeted to a compartment that is not in contact with the circulation may not be labeled. The same is true for cells that do not contact the circulation. Chemokines are notorious for binding multiple chemokine receptors (conventional and atypical chemokine receptors).

Selectivity of a chimeric chemokine needs to be demonstrated by assessing its binding profile versus atypical and conventional chemokine receptors. This is important, because conventional chemokine receptors may bind the chimera and contribute to the labeling pattern in vivo. Interpretation of data obtained with fluorescent chemokine chimeras is further complicated by receptor-independent binding such as binding to extracellular matrix.

The information provided in this report remains fragmentary. This is because the paper aims to map multiple receptors in multiple organs, which precludes in depth analysis. An exceptional resource would provide detailed information for each reporter for all relevant cell types in an organ of interest. This may be complemented by using fluorescent chemokines (standard and chimeric) in wildtype and receptor knockout mice. Knockout validation is necessary for each receptor to determine whether chemokine binding depends on the receptor of interest. In this context, quantitative analysis of the signal in wildtype and knockout tissues is strictly required. Results need to be presented in a tabular summary.

In sum:

The manuscript does not meet the strict criteria of a PLOS Biology Methods and Resources article.

Reviewer #2:

This extraordinary paper develops novel atypical chemokine receptor selective probes for elegant in vivo imaging to define the broad distribution of 3 receptors at the cellular level in a broad array of immune and other organs. The paper's scope aligns well with the Methods and Resources section of PLoS Biology. A problem in understanding the in vivo function of these receptors is the lack of suitable antibodies and receptor-selective chemokines. The authors have engineered highly selective chimeric chemokines to solve the latter problem. Moreover, this approach overcomes tissue autofluorescence attendant with GFP localization in some organs and GFP provides only cytosolic localization information. The group has worked on this approach in previous papers, but the current work is clearly the most advanced and expanded particularly with regard to the new ACKR GPR182. The methods are solid and the results are presented in great detail and described with utmost attention to clarity. Nevertheless, I do have some suggestions and questions to guide what I think are necessary revisions.

1. Title: Needs sharpening. 'Expression' and 'anatomical localization' are somewhat redundant, conditions are homeostatic, not inflammatory, and species is mouse.

We have modified the title as suggested by the reviewer.

ACKR should be switched to Ackr throughout the paper.

We thank the reviewer to the raising the issue. A requirement of a nomenclature consensus in writing is much appreciated. However, as we use ACKR referring to the proteins and not genes, we think that the proposed spelling style would be inaccurate as would allude to fish proteins.
We have consulted the guidelines @ Biosciencewriters.com
https://www.biosciencewriters.com/Guidelines-for-Formatting-Gene-and-Protein-Names.aspx
*"**Mice and rats**:* Gene symbols are italicized, with only the first letter in upper-case (e.g., *Gfap*). Protein symbols are not italicized, and all letters are in upper-case (e.g., GFAP)."
***Fish**:* In contrast to the general rule, full gene names are italicized (e.g., *brass*). Gene symbols are also italicized, with all letters in lower-case (e.g., *brs*). Protein symbols are not italicized, and the first letter is upper-case (e.g., Brs)."
We would appreciate a decision by the editor which style we should use.

2. Abstract: indicate the study was restricted to homeostatic conditions in young mice. Inflammatory conditions might yield different results. Line 40: Why were Ackr1 and Ackr2 omitted from the study (except for Figure 2 ex vivo)? Using the word comprehensive is awkward in this context.

We thank the reviewer for this comment. We have rephrased the abstract including the conditions of the mice used and removed "comprehensive". We mention the conditions of the mice also in the introduction (line 45/46 abstract and 134 introduction). In this study we focused on the atypical receptors 3, 4 and 5, receptors that interact with homeostatic chemokines (now mentioned in the Abstract and Introduction). ACKR1 is phylogenetically the most distant and appears to function differently (see below) and like ACKR2, interacts mostly with inflammatory chemokines.

3. Introduction: the description of the receptors is a bit confusing. The authors make the distinction that ACKR1 is a sink but not a scavenger unlike ACKR2 which is a scavenger, delivering chemokines for lysosomal degradation. Then refer to ACKR3 as a scavenger and a sink without

describing how it might be both. They should stick to biochemical functions or else define these metaphorical terms more exactly so there is no confusion.

We thank the reviewer to suggest clarification of the terms sink vs. scavenger. We changed the wording on line 77 to better outline the scavenging activity of ACKR2 and removed the term sink for ACKR3 (lines 94, 130).

Also there is no introduction to the beta arrestin signaling capacity of ACKR3 and its potential importance.

We thank the reviewer for the suggestion. We have added a reference, which suggests ACKR3 may signal through β-arrestins. However, for ACKR2-4 it was shown that signaling though β-arrestins is dispensable we added relevant references (lines 66-69).

The authors should indicate to what extent the CXCL12 binding function of ACKR3 has been shown to explain the cardiac valve stenosis seen in ACKR3 ko mice, and the evidence that it plays a role in HSC retention in BM niches.

4. Line 108: the paper in Molecular Cell on the mechanism of ACKR3 control of cardiac valve development also suggested an interaction with adrenomedullin.

In the paper describing the lethal phenotype (Sierro, PNAS 2007) occurring perinatally of mice with general or endothelium-specific ACKR3 deletion the authors reported that *Hbegf* and *Adm* were downregulated in the thickened semilunar valves (SLV) and speculated on an interplay of the genes with ACKR3. Later the group of K Caron (Klein, Mol Dev, 2014) proposed adrenomedullin as ligand for ACKR3 in lymphatic vessel formation in the heart including lymphatic hyperplasia and lymphedema. However, it was shown in another recent study including also the current corresponding author (MT) that ACKR3 expression is dispensable for postnatal lymphangiogenesis, lymphatic morphology and their drainage function (Sigmund et al. 2023). The following is *for the reviewers' eyes only:* Please see the abstract of a submitted manuscript by the same group, including the corresponding author of our manuscript (MT) reaching an unequivocal conclusion that adrenomedullin does not interact with ACKR3.: "*Atypical chemokine receptor 3 (ACKR3) is a scavenger of the chemokines CXCL11 and CXCL12 and of several opioid peptides. Additional evidence indicates that ACKR3 binds two other non-chemokine ligands, namely the peptide hormone adrenomedullin (AM) and derivatives of the proadrenomedullin N-terminal 20 peptide (PAMP). AM exhibits multiple functions in the cardiovascular system and is essential for embryonic lymphangiogenesis in mice. Interestingly, AM-overexpressing and ACKR3-deficient mouse embryos both display lymphatic hyperplasia. Moreover in vitro studies suggested that lymphatic endothelial cells (LECs), which express ACKR3, scavenge AM and thereby reduce AM-induced lymphangiogenic responses. Together, these observations have led to the conclusion that ACKR3-mediated AM scavenging by LECs serves to prevent overshooting AM-induced lymphangiogenesis and lymphatic hyperplasia. Here, we further investigated AM scavenging by ACKR3 in HEK293 cells and in human primary dermal LECs obtained from three different sources in vitro. LECs efficiently bound and scavenged fluorescent CXCL12 or CXCL11/12 chimeric chemokine in an ACKR3-dependent manner. Conversely, addition of AM induced LEC proliferation but AM internalization was found to be independent of ACKR3. Similarly, ectopic expression of ACKR3 in HEK293 cells did not result in AM internalization, but the latter was avidly induced upon co-transfecting HEK293 cells with the canonical AM receptors, consisting of calcitonin receptor-like receptor (CALCRL) and receptor activity-modifying protein (RAMP)2 or RAMP3. Together, these findings indicate that ACKR3-dependent scavenging of AM by human LECs does not occur at ligand concentrations sufficient to*

trigger AM-induced responses mediated by canonical AM receptors". Given these observations we prefer at this point to leave the discussion on a potential ACKR3/ADM axis out of the current manuscript focusing on the expression of ACKR3.

5. Line 117: ACKR5 implies that it does not activate a G protein. To what extent has that been investigated?

We thank the reviewer for raising this question. We refer now at line 126/127 to two previous publications indicating that GPR182 does not signal through G-proteins.

6. Line 317: this implies that there is such a thing as an arteriolar sinusoid.

We apologize for the confusion by inaccurate wording. Sinusoids are always of venous nature and rephrased the sentence accordingly (line 329).

7. Line 363: what are the ACKR4+ 'non-endothelial Lyve-1 and CD31 negative cells of the submucosa'?

We thank the reviewer for the comment. We added new data and rephrased the text to clarify. The cells are of a mesenchymal origin as they express vimentin (lines 379).

8. Line 501: what cells in the bone are expressing ACKR3?

We thank the reviewer for the comment. We now mention that the cells are "osteocytes" (line 524).

9. Line 502: why would scavenging CXCL12 help to retain HSCs in the niches, when CXCL12-CXCR4 signaling is known to be critical for retention?

We agree with the reviewer that the conclusion by Le Mercier et al. appears paradoxical, given the mobilization of stem cells with the FDA approved CXCR4 inhibitor AMD3100. However, Marin and Stumm have shown that high levels of endogenous CXCL12 can downregulate CXCR4. We discuss this now on lines 525-528.

10. The Discussion should integrate the findings with what is known about ACKR2, as the authors have done for ACKR1.

As suggested by the reviewer we discuss the role of ACKR2 in lymph nodes (lines 516-519).

11. I can't read the headings or the x-axis labels in figure 2D. Too small.

We thank the reviewer for pointing out the illegible axis labeling. We removed the headings because the content (receptor name) is repeated in large letters in the panels and increased the letter size of the x-axis.

Reviewer #3:

This methods and resources paper by Melgrati and colleagues describes the generation of a reporter mouse for the most recently characterised atypical chemokine receptor GPR182. These have been analysed in detail along with previously described reporters of the atypical chemokine receptors ACKR3 and ACKR4. In addition, a novel tagged chimeric chemokine (CXCL11_20) has been generated that appears to function as a specific target of GPR182. This has been used to trace functional GPR182 expression in vivo. Overall this paper adds to the reagent base available to study atypical chemokine receptors and provides the first clear picture of the expression profile of GPR182 in vivo. The expression of ACKR3 and ACKR4 described within are generally confirmatory of previous reports with some extension of the previous analysis. In general the experiments have been performed robustly and the imaging analysis presented is very impressive.

The following comments and questions should be addressed:

1) Figure 1: It would be of interest to examine ACKR4 and GPR182 sinusoids in the spleen using tissue clearing as has been done in ACKR3 mice in 1C.

We have now performed clearing of spleens from reporter mice (ACKR3/GPR182 and ACKR4/GPR182) and included them in Figure 1, panel D. ACKR3/GPR182 shows the expression of the receptors obtained by measuring over 400 Z-plains at 0.5 µm distance. The ACKR4/GPR182 spleen shows the expression (same acquisition settings) using 3D reconstruction (Imaris), which was technically not feasible for ACKR3/GPR182.

2) In Line 289 it is stated that the arrows in Fig2B right point to ACKR3 expression in large sinusoids. However, the arrows in this figure point to GPR182+ACKR3- cells? Please clarify.

We thank the reviewer for spotting this mistake. We have changed the arrows (Figure 1B) (white arrows) pointing now correctly on large vessels.

3) The visualisation of expression of ACKR3 in MZ B cells that is claimed is difficult to discern from the images in Fig1B. Can a closer up image of this be provided?

We apologize for the substandard visual discrimination of MZ B cells in the image. We have enhanced the contrast making the GFP positive MZ B cells stand out more and marked them with gray arrows.

It would also be of interest to look more closely at ACKR4 expression in the B cell compartment given previous reports. Ideally a FACS analysis of this should be done in the reporters too. Is GPR182 also expressed in B cell lineages?

The role of ACKR4 in B cells is controversial. The $ACKR4^{-/-}$ strain shows a hyperactivated B cell phenotype that is not observed in $ACKR4^{GFP/GFP}$ knock out mice used in our study. We mention this now in the introduction. As previously reported, ACKR4 was found on GC B cells (lines 104-107). At difference to ACKR3 and ACKR4, GPR182 is not present in the $CD45^{+}$ compartment of heathy mice. We show this in the new supplementary figure S3.

4) A FACS analysis of ACKR3 and ACKR4 in LN stroma should be presented (perhaps supplementary) to complement the FACS analysis of these cells with respect to GPR182.

We have added a new supplementary Figure S4 showing flow cytometry of ACKR3, ACKR4 and GPR182 expression in CD45 negative cells of lymph nodes (lines 333-334).

5) ACKR4 has previously been reported to be expressed in fibroblasts (Thomson et al. JI 2018; Bastow et al. PNAS. 2021). Whether the ACKR4+ cells in intestine (Fig6) co-express fibroblast markers should be tested.

We thank the reviewer for bringing up this point. We have added new panels to figure S5 showing the co-expression of the ACKR4 positive cells with vimentin and mention in the revised text their mesenchymal origin (lines 536/7).

6) Fig 7A: Does the difference in MFI of CXCL11_20 increase in GPR182 cells significantly increase from 4C to 37C? If not then this would indicate that GPR182 is mediating chemokine binding/sequestration rather than uptake. Please clarify.

We thank the reviewer for pointing this out. We have repeated the experiments (Fig 7A) and obtained clearer data, which show a marked difference in MFI between 4°C and 37°C consistent with binding at low temperature and uptake at 37°C.

7) Similarly, in 7E, how is CXCL11_20 uptake distinguished from binding in the spleen endothelial cells examined here?

We thank the reviewer for this comment. We changed the phrasing on line 915 replacing "uptake" with "binding and uptake" in the figure legend.

7) References 49 and 31 are duplications.

We thank the reviewer for spotting this mistake and we have also re-checked all references.

8) The methods states that sections were stained with anti-CD31-AF488 (line 195). It is not clear how this is spectrally distinguished from the GFP signal from the reporters that express GFP. Please clarify.

We apologize for the confusion regarding this image. We have now repeated the experiments using anti-eGFP antibodies counterstained with secondary antibodies labeled with AF488. Here, like in the figure used in the previous version, the discrete endogenous eGFP signal in the liver could not be visualized over the relatively high autofluorescence background, as now mentioned in line 460/3 and shown in Fig S7A. Therefore, amplification with an anti-eGFP antibody and secondary antibody was required to reveal the eGFP protein.

9) Line 72-73: ACKR2 is also able to scavenge CXCL10 so is not restricted to CC chemokines as implied here.

We thank the reviewer for pointing this finding out. We now mention in lines 77/8 that ACKR2 also interact with CXCL10.

10) Figure 7F should be referred to in the text of the paragraph starting on line 413

Thank you, now we refer to this Figure correctly (line 435).
